# Characterisation of AZ31 metal matrix composites reinforced with carbon nanotubes

**DOI:** 10.1038/s41598-023-44719-x

**Published:** 2023-10-18

**Authors:** Sandeep Ganesh Mukunda, Satish Babu Boppana, I. A. Palani, Samuel Dayanand, T. Aravinda

**Affiliations:** 1 Department of Mechanical Engineering, School of Engineering, Presidency University, Bengaluru, 560064 India; 2 Department of Mechanical Engineering, IIT Indore, Indore, 453552 India; 3 Department of Mechanical Engineering, Government Engineering College, Gangavathi, 583227 India

**Keywords:** Composites, Metals and alloys

## Abstract

The focus of this study revolves around the synthesis of AZ31 metal matrix composites (MMCs) reinforced with carbon nanotubes (CNTs) using the powder metallurgy technique. Various compositions of CNTs were incorporated into the AZ31 alloy matrix. The sintered specimens were analysed using microstructural characterization and Fourier transform infrared (FTIR) spectroscopy. Furthermore, differential scanning calorimetry (DSC) were conducted to investigate the impact of sintering on the processed composites. Corrosion studies were performed in a sodium chloride (NaCl) medium, and Tafel curves were plotted to assess corrosion behaviour. It was observed that composites enriched with 0.5 wt.% CNTs demonstrated the highest level of corrosion resistance among the synthesized AZ31 metal specimens.

## Introduction

The significance of two fundamental attributes in various engineering applications, particularly those pertaining to aerospace, defence, and transportation sectors, cannot be overstated: low weight and high strength (or specific strength). When it comes to fulfilling these requirements, magnesium (Mg) stands out. Not only is it lightweight, but it also possesses remarkable workability and damping properties, making it an ideal choice. Additionally, magnesium is fully recyclable, contributing to its overall appeal. However, it is important to acknowledge that magnesium does have some drawbacks in terms of its bulk characteristics. These include low stiffness, limited strength, and a susceptibility to corrosion, particularly in salty environments^1,2^.

In light of these considerations, researchers have directed their attention towards Metal Matrix Composites (MMCs). These innovative materials incorporate carbon-based nanomaterials as reinforcing agents, offering exceptional mechanical properties^1^. The utilization of lightweight materials is on the rise in the locomotive and space engineering due to the need for fuel efficiency and reduced CO_2_ emissions. Among these materials, magnesium and its alloys have garnered significant attention. With a density of 1.74 g/cm^3^, magnesium (Mg) stands out as one of the lightest structural metals^1–3^, making it a highly promising choice in sectors such as automobiles, aviation, and 3C (computer, communication, and consumer electronics).

However, despite its advantages, magnesium alloys face limitations in terms of stiffness and strength, which restricts their broader development and application. In comparison to steel and aluminium compounds, magnesium alloys exhibit significantly lower strength and stiffness. To overcome these limitations, researchers have explored the use of reinforcements for instance earthenware elements, carbon threads, and metal elements, along with various composite fabrication techniques, to enhance the strength of Mg alloys^4^. Nonetheless, magnesium falls short of aluminium when it comes to mechanical strength and corrosion resistance. The hexagonally closed packing (HCP) crystal structure of magnesium alloys impedes atomic slipping, thus limiting their deformation at normal temperatures. To meet the demand for low-weighing and high-strength materials, MMCs have emerged^5–7^. By incorporating reinforcing agents such as metal-based materials, ceramics, and carbon fibres, the strength of magnesium alloys can be further enhanced^8,9^. In particular, Multi-Walled Carbon Nanotubes (MWCNTs) have been successfully utilized to increase the mechanical properties of Mg-based composites. Magnesium-based alloys possess exceptional lightness, low density, high damping ratio, and a favourable weight-to-strength ratio^4^. However, their widespread use has been hindered by wear characteristics, low stiffness, absolute strength, and susceptibility to creep failure^10^. By introducing a small amount of reinforcing agents, the characteristics and applications of Mg-based alloys can be significantly improved. Recent advancements have focused on the incorporation of carbon nanotubes (CNTs) in magnesium-based composites, which have led to improvements in both mechanical and electrical properties. CNTs possess unique capabilities in terms of mechanical, electrical, and thermal properties, as well as low density, making them ideal for developing nanostructure materials^11^. In the realm of MMC reinforcements, CNTs have gained significant attention due to their ability to enhance strength, stiffness, and possess high strength (30 GPa) and stiffness (1 TPa)^12,13^. Numerous studies have explored the reinforcement of Al, Mg, Ti, and their alloys using CNTs^11,14,15^. Carbon Nanotubes (CNTs) are gaining popularity as reinforcing agents in Magnesium (Mg) alloys due to their unique properties, including their nanoscale dimensions, low density, and high stiffness^16–18^. When uniformly dispersed within magnesium matrix composites, CNTs contribute to the enhancement of their mechanical properties. By providing heterogeneous nucleation sites and acting as intermediaries for applied stresses, CNTs facilitate grain refinement and result in improved strength. In the case of aluminium composites reinforced with CNTs, they have been found to exhibit approximately twice the Young's modulus and tensile strength compared to the unreinforced matrix^19–21^. However, achieving uniform distribution of Carbon Nanotubes in the metal matrix during manufacturing CNT-reinforced MMCs remains challenging^22–24^. CNTs tend to aggregate due to strong Van der Waals forces, impeding uniform dispersion^1,2,9,15,16^. The control of bond between Carbon Nanotubes and the metal matrix is also difficult due to the wettability of carbon and metals, including magnesium^9,25–27^. While various methods such as stir melting, ultrasonic dispersion and chemical coating have been developed to disperse various reinforcements like CNTs in metal matrices^25,28^, achieving even dispersion throughout molten metals remains a challenge^13,29–31^. Some of these methods may also compromise the integrity of CNTs^9^. The accumulation of Carbon Nanotubes in the magnesium matrix composite can have adverse effects on the properties of the composites^32–34^.

The development of CNT-reinforced AZ31 magnesium MMC using an efficient powder metallurgy method is used in this study to produce magnesium alloys with the best possible mechanical properties.

## Experimental procedures

Raw multi-walled carbon nanotubes with 95% purity and AZ31 were used in this study without further purification. Multi walled carbon nanotubes as received were subjected through Transmission Electron Microscopy (TEM). In the fabrication process of the composite sample, the powder metallurgy approach is employed. Initially, AZ31 alloy in powder form is blended with varying weight proportions of CNT (0%, 0.5%, 1% and 1.5%) these are given symbolic names as given in Table 1. To ensure accuracy, a Shimadzu ATX224 weighing scale with a precision of 0.1 mg is utilized. The blending is carried out under precise conditions to prevent corrosion.

**Table 1:** The composition of various specimen prepared.

Description	Code
Pure AZ31 alloy	AZ31
0.5 wt.% CNT + AZ31 alloy	0.5 wt.% CNT–AZ31
1 wt.% CNT + AZ31 alloy	1 wt.% CNT–AZ31
1.5 wt.% CNT + AZ31 alloy	1.5 wt.% CNT–AZ31

**Figure 1:**
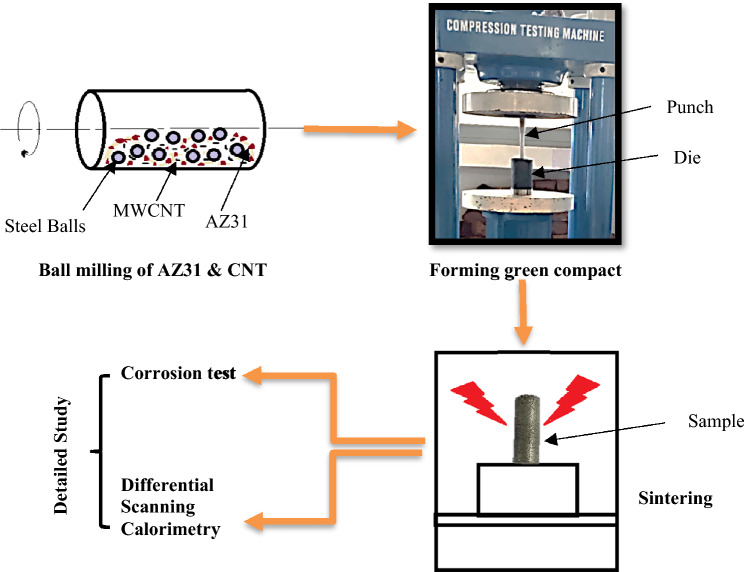
Flowchart of research work.

Figure 1 presents a step-by-step methodology employed in the current research.

Following the combination of powders, the mixture undergoes blending in a high-intensity planetary ball milling machine for a duration of 3 h. The milling machine operates at a rotational speed of 400 rpm, employing a 5 mm diameter treated steel ball as the crushing medium. The powder-to-ball ratio is maintained at one-tenth. To prevent overheating, the milling machine is periodically turned off for 16 min after every 40 min of operation. Subsequently, the blended powders were compacted into cylindrical billets with dimensions of 30 mm in diameter and 35 mm in length. This compression process is carried out using a universal testing machine, applying an external pressure of 600 MPa. To ensure the preservation of the sample's integrity and prevent any deterioration, particularly oxidation, the compacted billets were stored meticulously in a sealed box.

Finally, the prepared samples undergo the sintering process in an argon gas environment within a sintering furnace. The sintering is carried out for a duration of 2 h at a temperature of 535 °C^21,35^.

### Microstructural studies

After preparing the composites, polishing was done using a disc polisher and then proceeded to analyze them using various techniques, including image analysis, Scanning Electron Microscopy (SEM), and Transmission Electron Microscopy (TEM).

### Corrosion test

The alloy and composite were cut into 15 mm diameter, 10 mm thick corrosion test specimens. Before the corrosion testing, the composite surfaces were ground with 500 to 1200 grit papers and polished with 0–1-micron diamond pastes, respectively. For the potentiodynamic polarisation test, a 3.5% NaCl solution was utilised as electrolyte and the specimen were immersed in the solution for 60 min.

### DSC

In a nitrogen environment with 50 ml/min, 10 mg specimens were subjected to DSC (DSC, DSC-60 Plus, Shimadzu Corporation, Japan). The scanning temperature range was adjusted at 24–600 ℃, with continuous heating rate of 10 ℃ min^−1^.

### FTIR

The transmission FTIR spectroscopy observations were made with a Fourier transform spectrometer (Shimadzu, IRAffinity-1S) in the 400–4000 cm^−1^ wavenumber range.

## Result

### SEM, TEM and microstructure analysis

**Figure 2:**
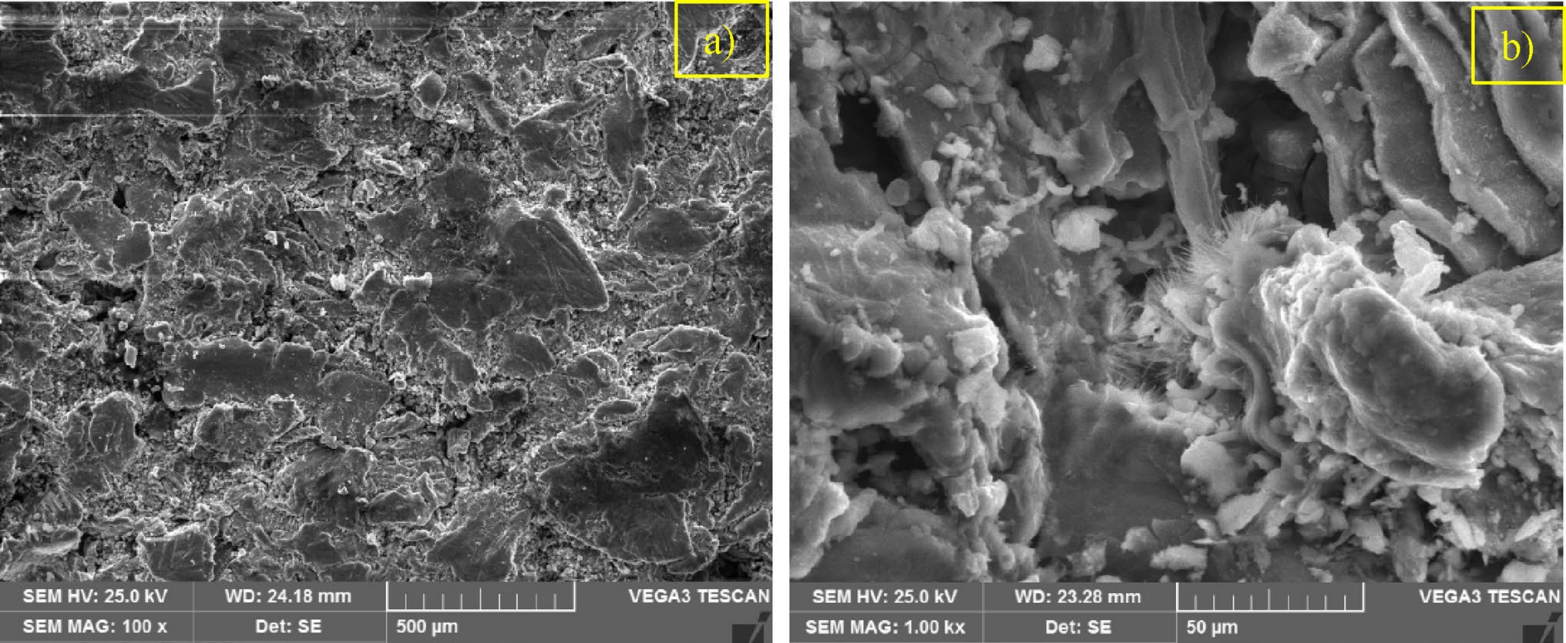
SEM micrograph of (**a**) AZ31 composite, (**b**) 1.5 wt.% CNT–AZ31 composite.

**Figure 3:**
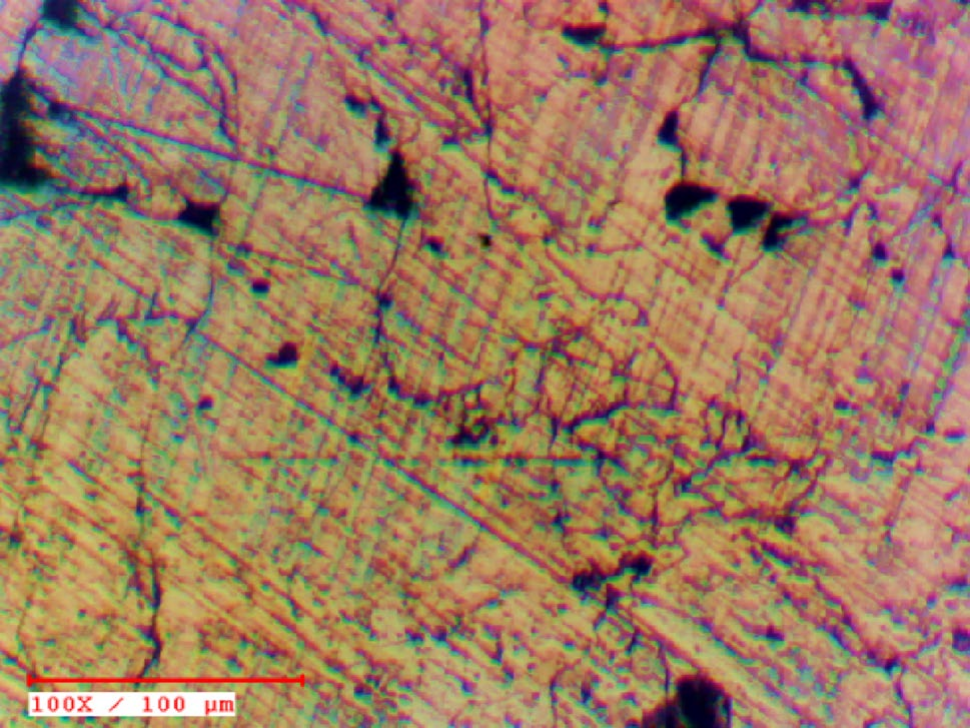
Optical micrograph of 1 wt.% CNT–AZ31 composite.

**Figure 4:**
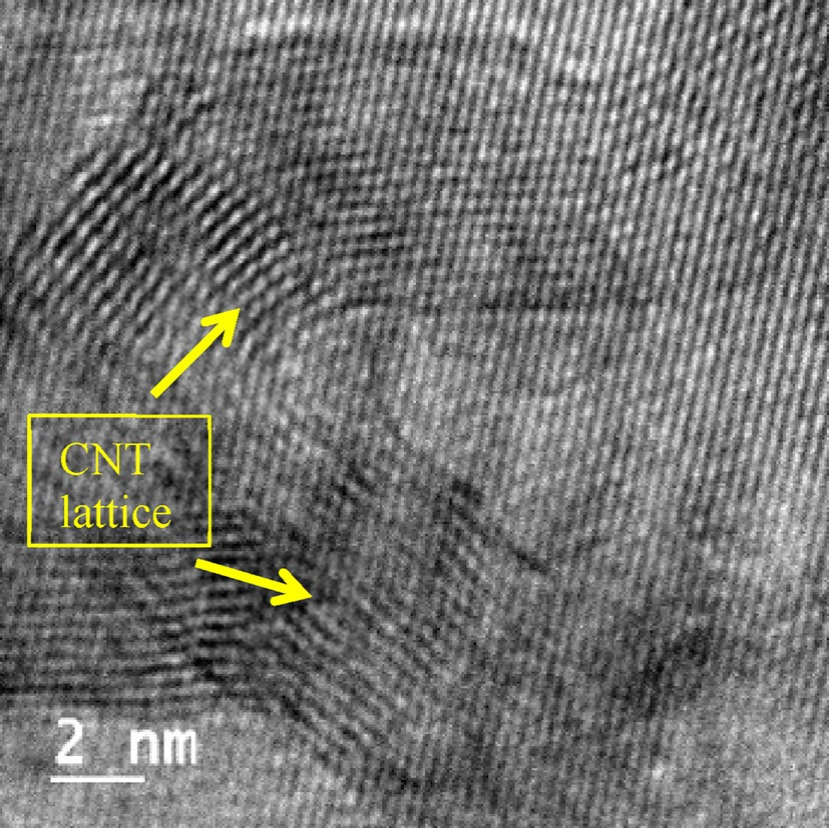
HRTEM of 0.5 wt.% CNT–AZ31 composite.

**Figure 5:**
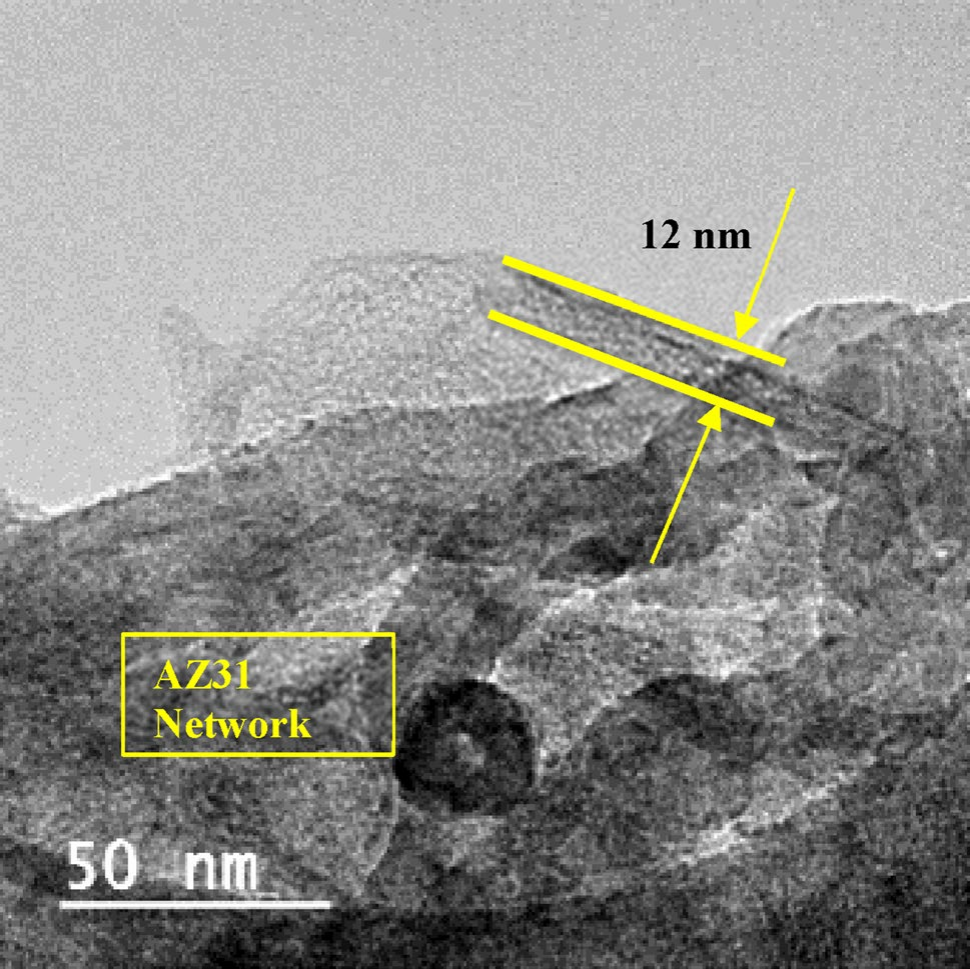
HRTEM of 0.5 wt.% CNT–AZ31 composite depicting CNT.

Scanning electron microscopy (SEM) was employed to investigate the microstructure and surface morphology of AZ31–CNT composite. The SEM analysis provided high-resolution images, enabling the examination of the sample's fine details and topographical features at a nanoscale level as shown in Figure 2. The micrographs obtained through SEM revealed the presence of grain boundaries, particle distribution, and potential defects. Figure 3 presents an optical micrograph illustrating the sintered AZ31 based composite with MWCNT. Figures 4 and 5 show TEM images of the prepared composite. Upon observation, it becomes evident that the majority of Carbon Nanotubes (CNTs) lattices are embedded within the β phases of the composites. It was observed in the 1 wt.% CNT–AZ31 & 1.5 wt.% CNT–AZ31 composite that CNTs in the composite appeared to be shorter than their original length, which was attributed to the significant ball milling and breakup that occurred during the Cold compaction process. This phenomenon aligns with the outcomes observed in other plastic deformation techniques^36,37^.

During the cold compaction, the shearing effect played a crucial role in cutting off the entangled CNTs, resulting in fragmented pieces. Subsequently, these fragments dispersed evenly into the Mg matrix due to the plastic flow of the magnesium. Despite the compaction process, the length of the CNTs in the composite still remained above 100 nm.

Figure 5 shows multi walled carbon nanotubes in AZ31 network that were subjected through Transmission Electron Microscopy (TEM), It is established that the average length is 100 nanometres and the diameter is 12 nm.

### XRD

In this research study, one of the sample consisting of 0.5% carbon nanotubes (CNT) and AZ31 alloy was investigated using X-ray diffraction (XRD) analysis.

**Figure 6:**
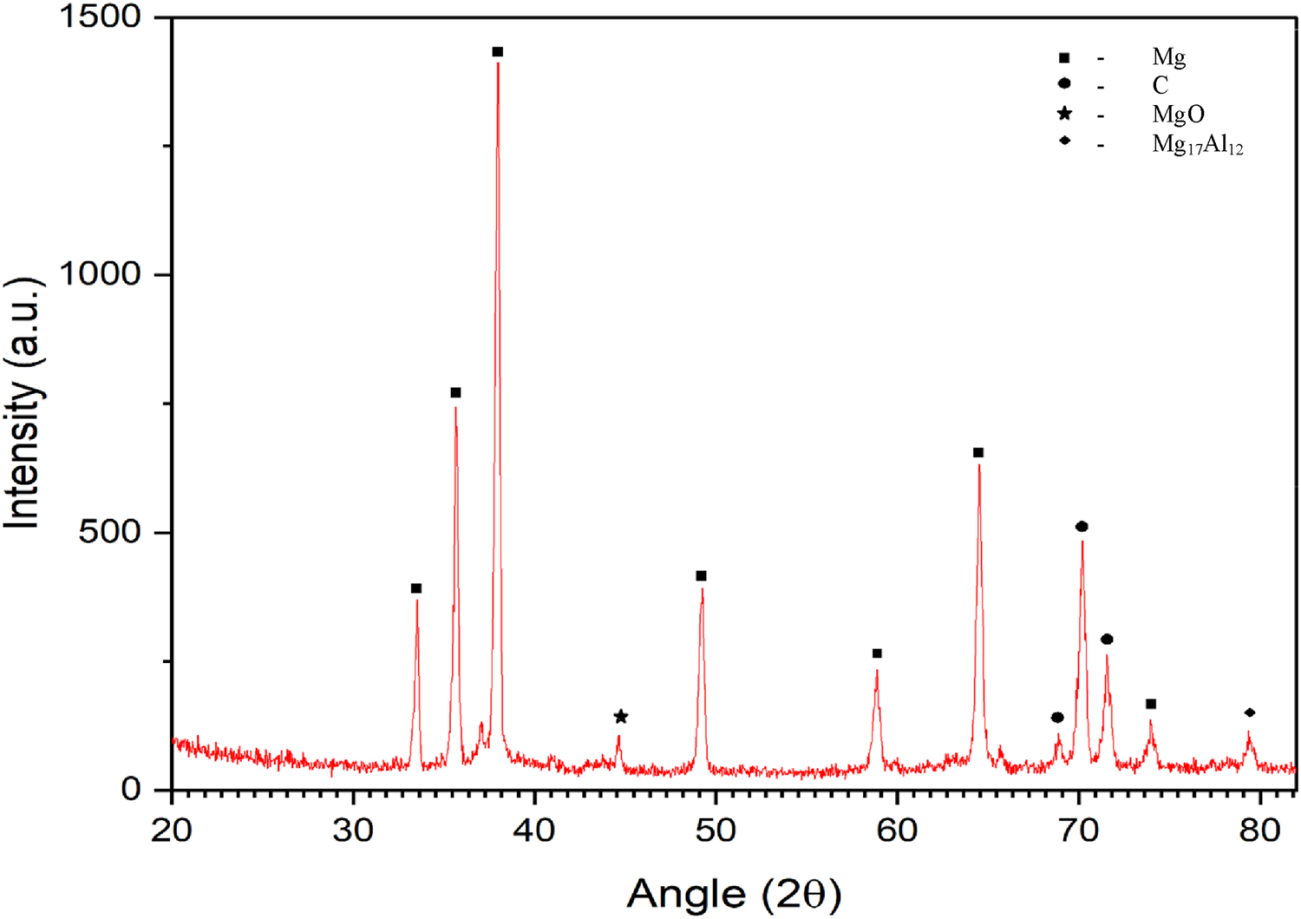
XRD of 0.5 wt.% CNT–AZ31 composite.

Figure 6 displays the X-ray diffraction (XRD) pattern of the 0.5 wt.% CNT–AZ31 composite sample. The presence of magnesium in the samples is clearly evident from the peaks observed. Additionally, there is a detectable carbon peak in all the samples, although its height is relatively small due to the limited amount of reinforcement (CNTs). Furthermore, the XRD pattern also reveals the presence of the aluminium-zinc (Al_0.5_ Mg_1.5_) intermetallic peak, which is consistently present across all the samples. The phase related to Mg–Al is due to the compacting and sintering process while preparing the composite. The presence of the MgO phase was consistently observed in all specimens reinforced with CNT. This occurrence is likely attributed to in-situ reactions between the magnesium (Mg) and surface oxygen contamination on both the metal powders and the carbon nanotubes (CNTs).

### EDS

**Figure 7:**
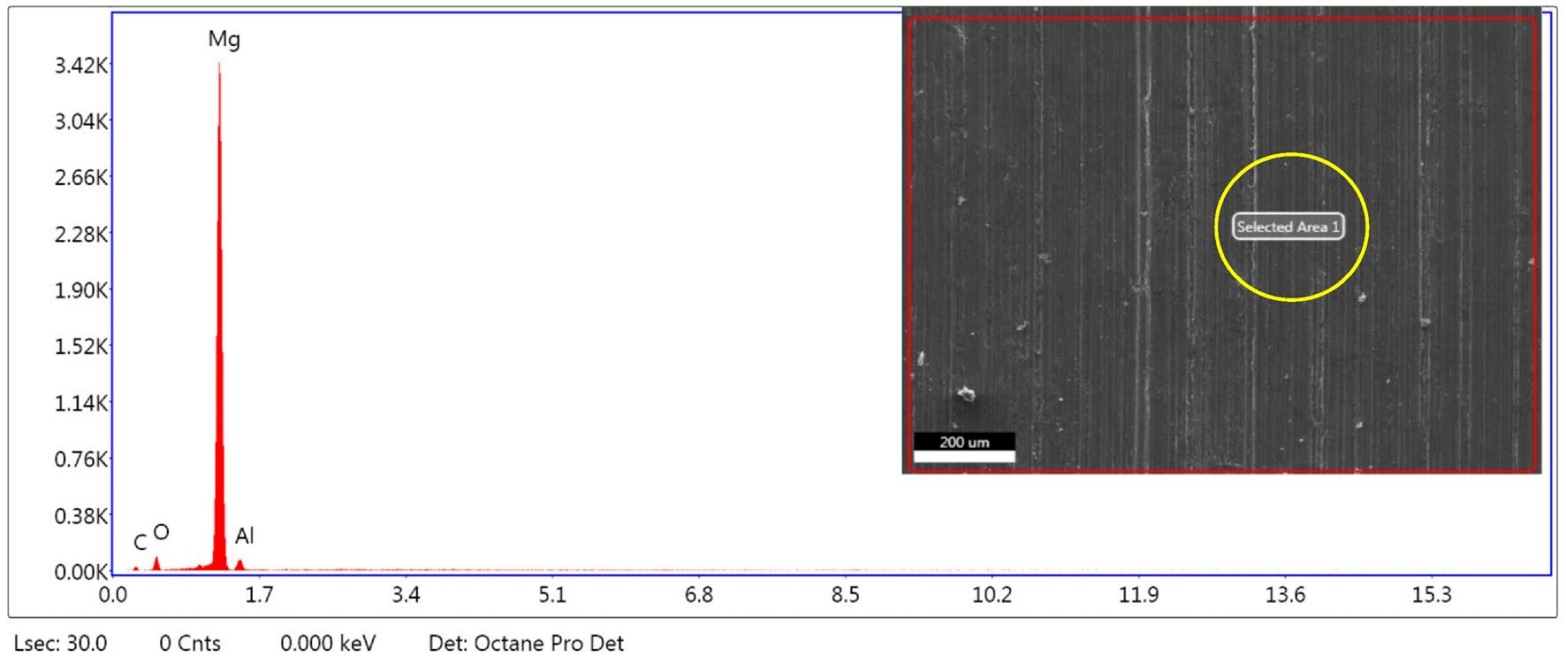
EDS of 0.5 wt.% CNT–AZ31 composite (inset: selected area).

The results of the EDS analysis confirmed the presence of both carbon and magnesium, which are the main constituents of CNT and AZ31, respectively. The carbon peaks indicated the successful incorporation of carbon nanotubes into the matrix, while the magnesium peaks represented the AZ31 alloy as shown in Fig. 7.

### Corrosion test

**Figure 8:**
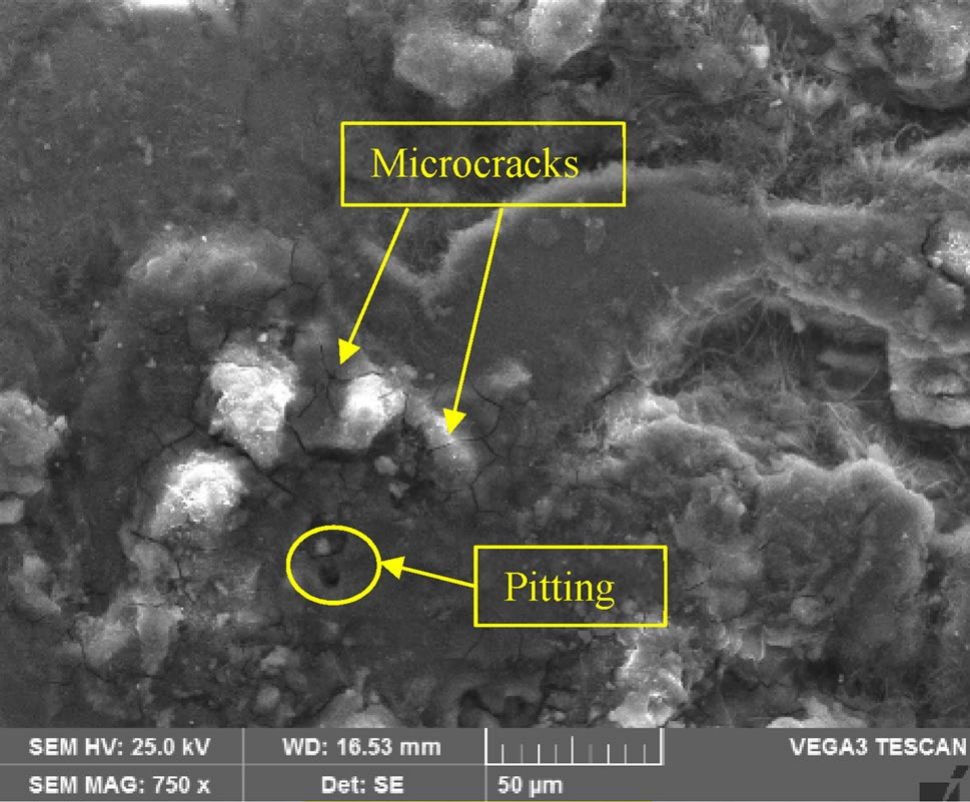
SEM micrograph of 0.5 wt.% CNT–AZ31 composite after corrosion test.

In Fig. 8, the observed white layer indicates the formation of an Mg(OH)_2_ layer on the surface. The scanning electron microscope (SEM) results revealed the presence of pits, which were formed due to the replacement of absorbed oxygen on the surface with Cl− ions from the solution. These Cl− ions, being relatively small in size, could penetrate through the developed oxide layer and replace the oxygen in areas where the metal-oxygen bonds are weakest^38^.

Moreover, in Fig. 8, microcracks can be observed, and these are attributed to an increase in the weight percentage (wt.%) of CNTs. This increase leads to the formation of clusters within the microcracks. Consequently, there is an increase in electron flow, leading to higher corrosion current density, which in turn leads to a higher corrosion rate. It is noted that 0.5 wt.% CNT–AZ31 composite provided the optimal corrosion resistance.

The findings of the conducted investigation indicate that composite with higher Carbon Nanotubes, specifically 0.5 wt.% CNT–AZ31 composite, exhibited superior corrosion resistance compared to structures containing higher wt.% of CNTs (e.g., 1 wt.% CNT–AZ31 & 1.5 wt.% CNT–AZ31 composite).

**Figure 9:**
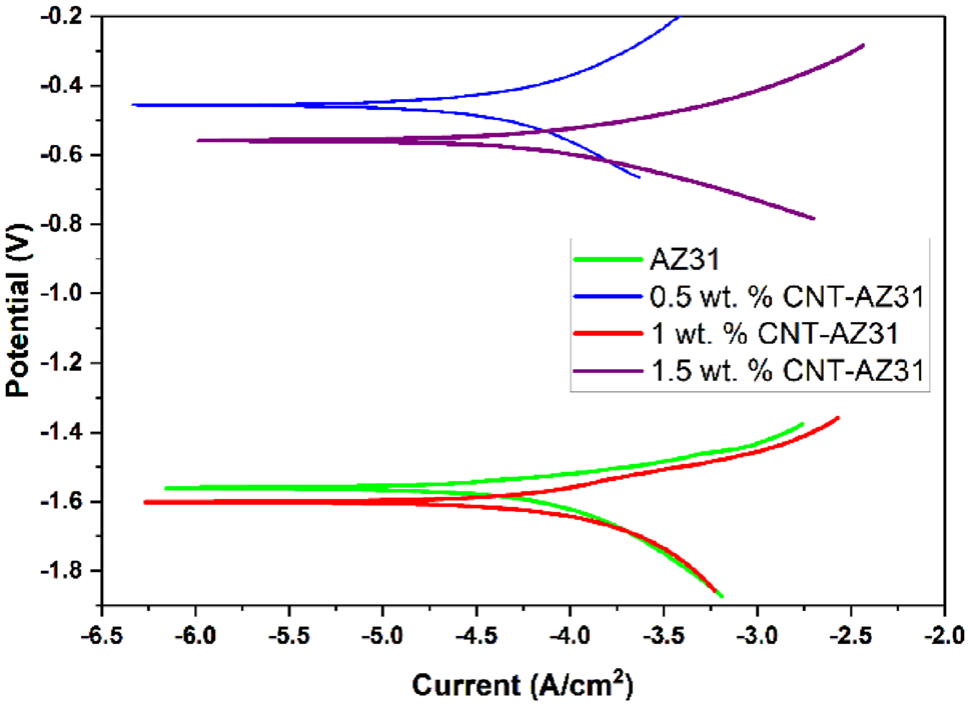
Potentiodynamic polarisation curves in NaCl solution for CNT–AZ31 MMC composites.

Figure 9 displays the plotted Tafel curve representing the selected sample. Upon referencing Table 2, it is evident that a higher ratio of MW carbon nanotubes (CNTs) in the Magnesium-based matrix leads to a reduction in corrosion rate for 0.5% CNT–AZ31, but increase in % of CNT beyond that resulted in increase in corrosion rate^39^.

**Table 2:** Corrosion properties of AZ31–CNT MMC and pure alloys calculated from potentiodynamic polarisation curves.

Matrix	CNT ratio (wt.%)	Corrosion rate (g/h)
AZ31	Unreinforced	2.212e−5
	0.5	2.138e−5
	1	3.078e−5
	1.5	3.054e−5

### Differential scanning calorimetry (DSC)

**Figure 10:**
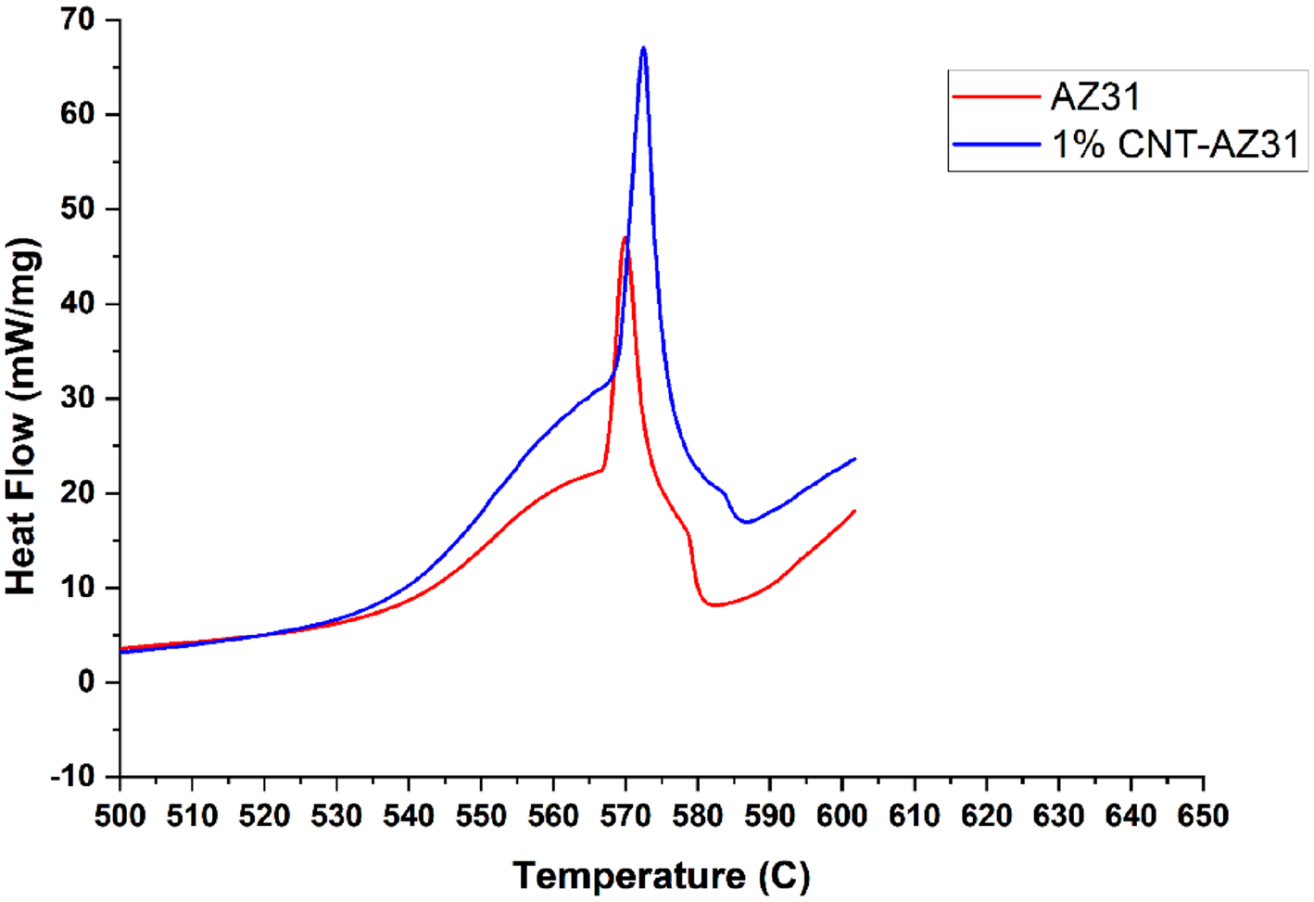
DSC curve of AZ31 alloy & 1% CNT–AZ31 composite.

DSC analysis of metal matrix composites (MMCs) reinforced with carbon nanotubes (CNTs), such as AZ31/CNT composites, provide useful insights into their thermal behaviour. DSC quantifies the heat flow associated with thermal transitions, enabling for the analysis of phase shifts, reactions, and thermal stability. The thermal behaviour of the AZ31/CNT composite was analysed using DSC data as shown in Fig. 10. The DSC curve exhibits the endothermic (heat-absorbing) and exothermic (heat-releasing) peaks related to the melting and crystallization behaviour of the AZ31 matrix. From the DSC analysis, for both composition (AZ31 and 1 wt.% CNT–AZ31 composite) the peaks occurred at temperatures of 570 °C and 574 °C but higher level of peak has been obtained in the 1 wt.% CNT–AZ31 composite with AZ31 composition and that determines the melting and crystallization temperatures and the corresponding enthalpies. The peak shifts in the DSC analysis could indicate interactions between the AZ31 matrix and the CNTs. These interactions influence the phase transitions or thermal stability of the composite.

### FTIR

**Figure 11:**
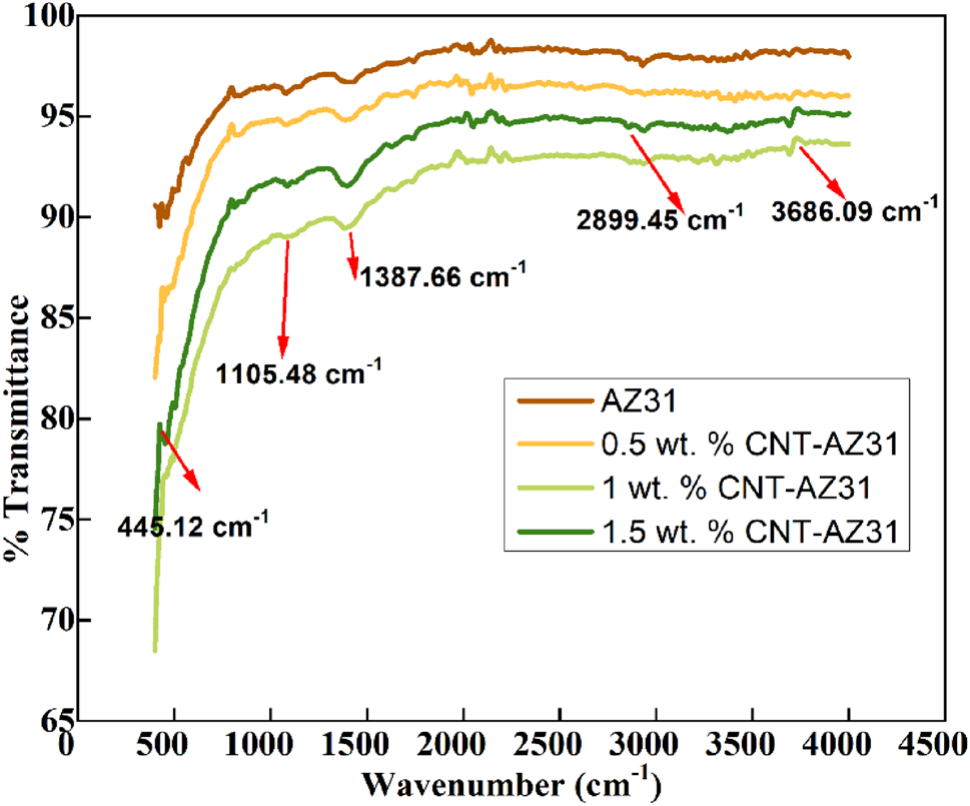
FTIR spectra of AZ31 composites.

The FTIR spectra of AZ31, 0.5 wt.% CNT–AZ31, 1 wt.% CNT–AZ31 & 1.5 wt.% CNT–AZ31 samples are shown in Fig. 11. The transmittance peak appearing at 3686.09 cm^−1^ were attributed to O–H stretching vibration and may be assigned to absorbed water molecules. The broad band at 2899.45 cm^−1^ is assigned to O–H stretching vibrations.

The peak at 445.12 cm^−1^ results from are attributed to metal-oxygen band the υ4 mode of O–P-bending, whereas the peak observed at 1105.48 cm^−1^ indicated the υ3 band of P–O stretching mode. The peaks approximately at 1387.66 to 1420 cm^−1^ correspond to the υ3 vibration mode of carbonate incorporated in the samples^40^. The broad absorption band at 1387.66 cm^−1^ were ascribed to N–O stretching vibrations. The obtained result is in good agreement with previous reports of IR analysis of samples^40,41^. Based on above spectra, if percentage of CNT would increase, the intensity of peaks would also increase.

## Discussion

In this research paper, the development of CNT-reinforced AZ31 magnesium metal matrix composites (MMCs) using an efficient powder metallurgy method was investigated to enhance the mechanical properties of magnesium alloys. The study is aimed to address the limitations of magnesium alloys, such as low stiffness and strength, by incorporating carbon nanotubes (CNTs) as reinforcing agents.

The experimental results revealed that the CNTs were well integrated into the magnesium alloy matrix, as observed through scanning electron microscopy (SEM) analysis and TEM studies. The corrosion resistance of the composites was significantly improved with the 0.5 wt.% CNT–AZ31 composite. This finding suggests that CNT-reinforced composites have the potential to inhibit corrosion processes in magnesium alloys.

EDS studies revealed the presence of constituents of the alloy and reinforcement justified the successful syntheses of composite.

The FTIR analysis confirmed the presence of various vibrational modes associated with CNTs in the composite material. Additionally, transmission electron microscopy (TEM) provided visual evidence of the bonding between CNTs and the AZ31 matrix, indicating the successful dispersion of CNTs within the magnesium alloy.

The DSC analysis revealed the information about peak shifts during the formation of alloy and composite. The higher level peak would relate to improved thermal stability.

These findings in CNT-reinforced magnesium alloys have promising implications for various engineering applications in sectors such as aerospace, defence, and transportation, where lightweight materials with high strength are in demand.

CNTs were successfully reinforced into the AZ31 matrix to produce composites. Moreover, the observed improvements in corrosion resistance, as well as the insights gained from DSC and FTIR analyses, contribute to a deeper understanding of the CNT-reinforced magnesium alloys. The practical implications of this research are significant, as it opens doors to the development of lightweight materials that can find applications in industries where the demand for fuel efficiency, reduced CO_2_ emissions are of prime importance.

## Data Availability

The data that support the findings of this study are available upon reasonable request. Requests for data should be directed to the corresponding author.
